# Cognitive strengths in first episode psychosis: a thematic analysis of clinicians’ perspectives

**DOI:** 10.1186/s12888-021-03627-y

**Published:** 2021-12-08

**Authors:** Peter Steele, Nicholas Cheng, Lisa J. Phillips, Shayden Bryce, Mario Alvarez-Jimenez, Kelly Allott

**Affiliations:** 1 Melbourne School of Psychological Sciences, The University of Melbourne, 35 Poplar Road, Parkville, VIC 3052 Australia; 2 Orygen, Parkville, Victoria Australia; 3 Centre for Youth Mental Health, The University of Melbourne, Melbourne, Australia

**Keywords:** Cognition, Psychosis, Youth, Recovery, Qualitative, Functional outcomes

## Abstract

**Background:**

Cognitive deficits are associated with poor functional outcomes in individuals recovering from a first episode of psychosis (FEP). Existing treatments that target cognitive deficits in FEP may enhance cognitive function, but improvements to real-world functioning are less consistent. Furthermore, these treatments may not adequately address the personal recovery goals of young people attending FEP services. A novel cognitive strengths-based approach may overcome these shortcomings.

**Methods:**

This qualitative study used semi-structured interviews to explore clinicians’ (*N =* 12) perspectives toward the potential development of a cognitive strengths-based assessment or treatment in FEP. The interviews were analysed using thematic analysis.

**Results:**

Five higher-order themes emerged: (1) pro-strengths attitude despite unfamiliarity and minimal use, (2) default to a cognitive deficit lens, (3) potential benefits of a cognitive strengths approach, (4) potential risks and barriers, and (5) considerations for successful implementation. While clinicians acknowledged their current deficit approach, they supported implementing a cognitive strengths assessment or treatment and highlighted their potential benefits for the personal recovery needs of young people with FEP.

**Conclusions:**

These findings suggest that a deficit-focused approach to cognitive function amongst clinicians may be common practice in FEP services. Nevertheless, a cognitive strengths approach was viewed favourably by clinicians and may represent a novel method of supporting personal recovery. Thus, the design and implementation of a cognitive strengths approach may be worthwhile. Future exploration of other stakeholder perspectives, such as young people with FEP, is essential.

**Supplementary Information:**

The online version contains supplementary material available at 10.1186/s12888-021-03627-y.

## Background

Long-term functional impairment persists in more than two-thirds of young people recovering from a first episode of psychosis (FEP) [1]. These young people may experience enduring functional disability affecting critical life domains (e.g., establishing relationships, developing independence) despite symptom remission [2, 3]. As core features of psychosis, cognitive deficits manifest before and remain after a FEP [4]. These deficits have shown to predict long-term functional outcomes in FEP more strongly than positive symptoms [5, 6]. Thus, cognitive impairment is a primary treatment focus for functional recovery in young people with psychosis [7].

Cognitive remediation (CR) therapies are an evidence-based treatment focused on enhancing cognition, with the ultimate goal of translation to functional outcomes [8, 9]. Meta-analyses show that CR is an effective method of improving cognition and functioning in adults with schizophrenia [9, 10]. Conversely, the impact of CR on cognition and everyday function in people with an early course of illness is significantly less compelling, with evidence of small treatment gains that are often inconsistent [11, 12]. Based on the rates of non-consent and attrition in RCTs [12], not all people with FEP want to engage with CR, suggesting that existing treatments may not engage some young people with psychosis. Treatment engagement is important given that it is linked to better outcomes and disengagement from FEP services is associated with a higher risk of relapse and poorer long-term outcomes [13, 14]. Thus, exploring alternative approaches that may enhance engagement and functional recovery in young people with FEP is worthwhile.

A complimentary cognitive strengths-based approach may enhance service engagement and functional outcomes in FEP by targeting positive psychological factors such as motivation [15], self-efficacy, and social relatedness [16]. This recently proposed paradigm strives to divert clinical attention away from ‘repairing’ deficits toward identifying and enhancing young people’s inherent strengths [6]. There is promising evidence that psychosis treatments adopting positive psychology principles enhance well-being and quality of life [17], are feasible and engaging [18], increase motivation, self-efficacy, and positive emotion [19, 20], and even improve vocational recovery and reduce hospital admissions and visits to emergency services [21]. A cognitive strengths-based approach to assessment and treatment may yield similar benefits in young people with FEP and should be further explored [6]. To our knowledge, there are currently no specific assessment measures or treatment methods focused on perceived cognitive strengths in the field [6].

Qualitative investigations are a valuable preliminary step in exploring new concepts [22] and developing novel treatments. They can provide a nuanced illustration of how cognitive strengths are conceptualised and perceived amongst stakeholders within the FEP service structure [6]. A recent qualitative study revealed that expert researchers (in the field of cognition and psychosis) mainly held positive attitudes toward the concept of focusing on cognitive strengths in FEP treatment, but also cautioned the potential to invalidate young people’s concerns, particularly their experience of cognitive deficits [23]. This research provided preliminary support from knowledge experts for the consideration of cognitive strengths as part of future assessment and treatment in FEP.

As a logical next step, the current study explored the perspectives of clinicians who work directly with young people with FEP (treatment experts) regarding cognitive strengths in FEP settings. It was envisaged that mental health clinicians would likely be acutely aware of the immediate treatment needs of young people experiencing psychosis and service-level implementation considerations. Thus, we aimed to gather clinicians’ insights regarding the early-phase conceptualisation of cognitive strengths, the perceived risks and benefits of focusing on strengths, and the practical considerations for developing a strengths-based assessment and treatment in FEP.

## Methods

### Setting and participants

Purposive sampling was used to recruit medical and allied health clinicians employed at the Early Psychosis Prevention and Intervention Centre (EPPIC) and who provided direct care to young people with FEP. EPPIC is a tertiary mental health service at Orygen Youth Health (OYH) that treats people aged 15–25 who have experienced FEP and reside in the north-western region of Melbourne, Australia. In total, twelve clinicians who were employed at EPPIC in the year 2018 were recruited.

### Procedure

Research ethics approval was obtained from the Melbourne Health Human Research Ethics Committee (HREC 2016.313). All methods were performed in accordance with the Declaration of Helsinki and the National Health and Medical Research Council of Australia’s *National Statement on Ethical Conduct in Human Research.* The principal researcher, who was not associated with EPPIC and was unknown to prospective participants, provided a brief study description to clinicians during clinical review meetings across EPPIC sites. Clinicians who expressed interest were formally invited to participate and provided written and informed consent. As recruitment progressed, clinicians from disciplines yet to be represented were selectively targeted to ensure the final sample reflected the multidisciplinary nature of teams at EPPIC (e.g., medical, nursing, psychology, and occupational therapy).

### Data collection

A semi-structured interview comprising sixteen pre-determined questions was developed for this study [see Additional file 1]. The interview questions primarily addressed perspectives toward cognition, cognitive strengths, applying cognitive strengths in the FEP context, incorporating strengths or cognitive strengths in current practice, considerations for potential future implementation, and potential risks and benefits of a cognitive strengths-based approach. All interviews were conducted face-to-face by author PS and ranged from approximately 25 to 40 min in length. Responses to interview questions were probed for clarification and further exploration. All interviews were audio-recorded and transcribed orthographically. Hand-written notes were compiled during each interview as part of a reflexivity journal to document initial observations and reflections.

### Data analysis

The data were analysed using thematic analysis according to Braun and Clarke’s [24] six-phase method. Data familiarisation was achieved by transcribing and thoroughly reviewing each interview. Manual coding was completed using an inductive approach as the study aims prioritised clinicians’ perspectives. Deductive methods were used to determine the aspects of the data’s semantic content that were relevant to the research focus. The codes from each interview were organised and refined using NVivo data coding software. Nine of the twelve interviews were independently coded by two research supervisors and an experienced co-researcher. Inter-rater agreement was achieved by reviewing discrepancies until a mutual consensus was reached. The final coding structure was developed through an iterative process of consolidating and revising codes. Recoding was completed as necessary according to the revised coding structure to ensure consistent coding across all interviews [25]. Codes were then sorted to candidate themes and explored using thematic maps. Finally, themes were refined by reviewing the extracts collated within each candidate theme to determine whether they were internally coherent and representative of the dataset.

## Results

### Participant characteristics

Twelve EPPIC clinicians (*M*_*ag*e_ = 33.33 years, *SD* = 8.39, range: 26–57; 66% female) were recruited from four disciplines: clinical psychology (*n* = 7), psychiatry (*n* = 2), mental health nursing (*n* = 2), and occupational therapy (*n* = 1). Three participants were investigators on research studies that addressed cognition in FEP. None of the participants had experience in research or clinical practice focusing on a cognitive strengths-based approach. The clinical experience of participants in an FEP-specific context ranged from 1 to 25 years (Mean = 5.79 years, *SD* = 6.6).

### Thematic structure

**Fig. 1:**
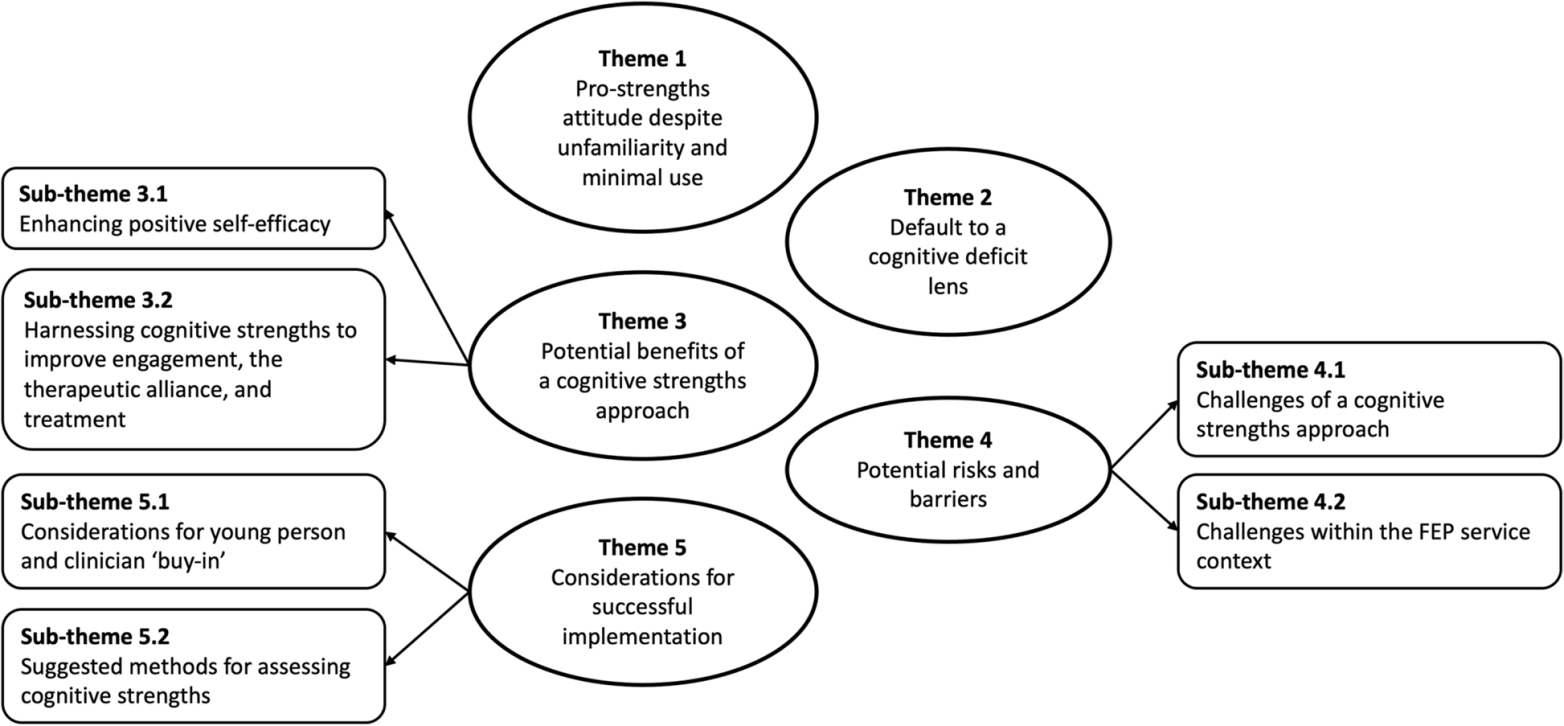
Thematic map

Five over-arching themes were identified, with three containing sub-themes. A thematic map is shown in Fig. 1. Using the semi-structured interview questions, no new themes emerged by the end of the twelve interviews.

### Theme 1: pro-strengths attitude despite unfamiliarity and minimal use

Clinicians generally held a positive stance toward strengths-based approaches in treatment. They believed it was important to consider young people’s strengths and incorporate them into their practices: *“If we’re gonna … assess what’s not going so well for the client, it’s just as important to focus on and highlight what they’re doing well”* (P2). Most clinicians seemed to engage with broader strengths in their current work and many also indicated that the early psychosis service (EPPIC) endorsed a recovery-oriented strengths-focused approach. Nevertheless, most clinicians recognised the minimal application of strengths in current practice and believed there should be increased attention toward young people’s strengths: “*...there should be more focus on strengths, and … I think young people would really appreciate that*” (P8); “*Again, we probably just don’t think about that [cognitive strengths] kind of enough*” (P9).

Despite the positive attitudes toward strengths approaches broadly, conceptualising cognition through a strengths-based lens was difficult and unfamiliar to clinicians: “*like, how do we...is there a consistent way of conceptualising what [a] cognitive strength is?*” (P5). The definitions were varied and consisted of multiple definitional frameworks [See Additional file 2]. Several clinicians defined cognitive strengths in relation to functional abilities: “*whether they [young people] could sort of...make a well-informed decision*” (P5); or whether young people can benefit from treatment, e.g., “*whether they are able to engage in actual cognitive therapy or not*” (P7).

Most clinicians recalled instances where they acknowledged young people’s character strengths (e.g., perseverance, resilience). Yet, they admitted that their involvement specifically with cognitive strengths was either minimal or non-existent. Those with minimal involvement did so indirectly when broadly considering other functional strengths: “*I don’t know that I would specifically home in on cognitive strengths...but, I guess, when I’m working with people, I would try to identify what their strengths are in any kind of field*” (P3).

### Theme 2: default to a cognitive deficit lens

Clinicians frequently and inadvertently resorted to a deficit-focused perspective during their interviews. For some clinicians, this occurred immediately when they were asked about cognitive strengths:

*“It’s really that perspective taking, the stuff that I’ve noticed that they can struggle with, and then like as I’m saying all this, I notice that I’m talking about it from a deficits place still.”* (P11).

More commonly, an initial reference to strengths transitioned to deficits:

“*So … time is important for vocational functioning. Like, knowing when to get to a certain place and have an idea of how long it’s gonna take to get there. That sort of, I suppose, forward-thinking and problem-solving. Um, I know for a lot of the clients that I work with, a difficulty is not being able to problem-solve, particularly if they’re using, like, public transport.*” (P2).

Some clinicians held a subtle deficit orientation by defining cognitive strengths as the resolution of a young person’s difficulties:

*“ … you know, using it [cognitive strengths] in your mental status … if someone is having difficulty keeping up with things in the session and that kind of stuff; but you know, you might reflect on those things improving if someone’s getting better but it’s still from a kind of deficit model rather than a strength model.”* (P6).

Several clinicians were aware of their own deficit focus and the deficit orientation of the FEP field in general: “*I would probably think more of people’s cognitive difficulties than cognitive strengths. Yeah, I think I probably would have more of a bias towards that*” (P7). Several clinicians added that the medical model remained an integral part of assessment and treatment in FEP: *“It’s harder because I suppose when we do cognitive assessments, it’s more sort of to look for deficits than strengths*” (P2).

### Theme 3: potential benefits of a cognitive strengths approach

#### Enhancing positive self-efficacy

Most clinicians believed that a cognitive strengths-based assessment and treatment could potentially enhance a young person’s self-efficacy. For example, one participant suggested that self-awareness and directed use of cognitive strengths could improve a young person’s self-confidence:

*“ … well, they’re really good at organising. Then you know, when they’re – let’s say at the school, that we sort of have programs where teachers can get them to do more sort of in-house sort of organisation for other people or activities. And that could really build on their self-confidence and self-esteem.*” (P5).

Other clinicians described positive effects on young people’s sense of empowerment and agency:

“*People would have...more of an awareness of areas that they can feel a sense of competency...and that can also then help with motivation … and a sense of agency because they’ve been able to think about the things that they can do.*” (P10).

Another participant similarly described cognitive strengths as a way for young people to, “*...start taking charge of their own healthcare and feel empowered that way*” and “*use those strengths to get [young] people to make their own decisions...with their own lives*” (P6).

This approach was thought to potentially help change the narrative around a young person’s treatment to incorporate their cognitive strengths:

“*...if we can embed some of the strengths of that client with their cognition into sort of a story that we’re developing with them, I think it helps sort of raise the awareness of what they’re doing well.*” (P2).

Clinicians believed that helping young people change their clinical narrative may alleviate their self-stigmatisation and perceived defectiveness.

#### Harnessing cognitive strengths to improve engagement, the therapeutic alliance, and treatment

Most clinicians believed that utilising a cognitive strengths approach would resonate with young people’s goals and priorities and therefore enhance engagement with the service:

*“I think when you point out someone’s strengths rather than pointing out their flaws and what we need to improve, and if you help them use their strengths, you know, you tend to get better engagement. You get better results because people feel empowered.*” (P6).

As such, clinicians commented that a cognitive strengths approach could enhance the therapeutic alliance, as they can acknowledge the young person’s attributes beyond their difficulties:

“*I guess it would make them hopefully feel like we’re not just viewing them as their difficulties or their problems...*” (P7).

Clinicians also highlighted the benefit of developing a richer understanding of a young person’s cognitive strengths. Awareness of cognitive strengths “*could help with formulation and understanding the person*” (P4) and subsequently inform how clinicians and young people can incorporate these strengths in their treatment: “ *… when you’re starting to focus on recovery, then identifying strengths that you can use within the therapy … would be really helpful*” (P3). This deeper understanding was also thought to assist them in determining what types of treatments would be possible or appropriate: *“ … it’ll give an indication of someone’s abilities, which will indicate what level of psychotherapy they’re capable of doing at the moment”* (P9). Thus, several clinicians believed that this knowledge could help them adopt the most suitable communication strategy when delivering treatment: *“if you found that someone was better at communicating visually, then you might use drawings or diagrams more in your therapy”* (P3). Clinicians also suggested that other professionals (e.g., teachers) can use this knowledge to maximise a young person’s vocational and educational recovery.

### Theme 4: potential risks and barriers

#### Challenges of a cognitive strengths approach

Clinicians were concerned that a cognitive strengths approach could reduce attention toward deficits or acute needs. If therapeutic time was made available for assessing and working with cognitive strengths, clinicians may *“end up not really focusing on what’s not going so well”* (P6) and *“almost ignore the deficits”* (P1)*.* Similarly, focusing on cognitive strengths was thought to interfere with risk assessment or management of acute psychotic symptoms. Nevertheless, several clinicians believed that young people’s difficulties would not be neglected given that the prevailing focus was on deficits.

Clinicians were further concerned that a cognitive strengths approach would engender unrealistic hope for young people. This could occur when few, or fewer than expected, cognitive strengths were found or where*, “the strengths that you find may not be the strengths that young people want to have in their brain”* (P8). Clinicians also indicated that false hope could arise if cognitive strengths identified during recovery regressed during relapse. Some clinicians felt that this situation could cause young people to experience a greater sense of loss: *“and that might, if it didn’t go well, that might bring about a sense of hopelessness”* (P9).

Clinicians were also concerned that focusing on cognitive strengths would risk invalidating young people’s concerns:

“*If you talk too much about strengths, it can be perceived as dismissing, like, ‘things are really shit for me right now and we’re talking about what’s...what I’m doing right’?*” (P1).

#### Challenges within the FEP service context

Time and resource limitations were frequently highlighted as a barrier to cognitive strengths assessment and treatment. Most clinicians believed it would be difficult to implement new assessments with their current time constraints:

“*I think a big factor for a lot of clinicians is the time constraints that holding an assessment sort of has on your demands*” (P2); “*The biggest thing is time, really.*” (P6).

Some clinicians were concerned about implementing a cognition-specific treatment, due to the variability in perspectives and experience in working with cognition across the early psychosis multidisciplinary teams. Others expressed related concerns that any new assessment would need to involve significant training of service staff. To address both the time and training/resourcing concerns, many clinicians indicated a preference for any cognitive strengths assessment to be outsourced to a specialist clinician or built into the service’s intake processes.

Clinicians also raised concerns that additional cognitive strengths assessments would increase the ‘assessment burden’ on young people who attend the service. For example, if young people undergo “*a good three or four assessments even before you kind of start the work properly*” (P4), a requirement to complete a cognitive strengths assessment might make the person “*feel like they’re just doing these endless assessments*” (P9).

### Theme 5: considerations for successful implementation

#### Considerations for young person and clinician ‘buy-in’

Clinicians believed that clear communication of the rationale of a cognitive strengths assessment or treatment was essential to gain ‘buy-in’ from young people: “*I think you’d have to explain it really, really well and it’d have to be quite transparent with why you’re doing it, what it means, and why it could be beneficial*” (P4). Clinicians urged that young people would need to believe that “*there was something tangible at the end of it”* (P9), *“otherwise they won’t do it, or won’t agree to do it*” (P8). Emphasising a clear benefit also addresses the time and resourcing issues identified previously: “*[the assessment or treatment] has to be more valuable...than the thing that they’re not doing*” (P9) and thus, “*It would need to be useful...like it would need to actually serve a proper function*” (P4). To reduce young people’s negative expectations from previous assessment experiences, several clinicians highlighted that the rationale should clearly state that the assessment *“isn’t about finding something that’s wrong, it’s about finding, you know, actually what you’re good at*” (P6).

Clarity in content and delivery of the assessment or treatment was deemed necessary for optimal engagement. Clinicians proposed avoiding jargon: “*if we’re going to stick with words like cognition and cognitive, you have to be able to also provide quick explanations of what those words mean*” (P1); “*I think as soon as you say cognitive strengths you’ve probably lost them a little bit*” (P4). To address these issues, several clinicians suggested including scenarios or examples throughout assessment delivery, both to clarify meaning and to help young people resonate with assessment items:

“*If they can draw on personal experiences somehow with a question that identifies oh, actually that was a strength to be able to do that, I think that would help personalise it rather than it being this kind of assessment of a list of strengths that they can’t relate to I guess*” (P10).

Several clinicians suggested using technology to enhance engagement: “*iPad stuff – they’re always more likely to be on board with*” (P6) and “*I think in particular for young people now … just the kind of paper copy versus like actually having an app where you can, you know, interact with an app I imagine would be a lot easier for them*” (P10).

Finally, several clinicians emphasised that it was important to, “*tell people you’re going to be able to give them feedback*” (P3) and that when provided it should be, “*Quick feedback that’s relevant*” (P9) and in a usable form.

#### Suggested methods for assessing cognitive strengths

There was no consensus for a particular assessment method. Most clinicians suggested that young people would respond negatively to traditional forms of assessment (e.g., self-report questionnaire): “*I think filling out any questionnaire, young people are sort of over it*” (P6). Nevertheless, some clinicians believed that it would be difficult to objectively measure a young person’s cognitive strengths without some form of standardised neuropsychological assessment. Thus, several clinicians accepted that traditional assessments might be suitable, but should be translated to a more youth-appropriate form.

Some clinicians stated that gathering information on cognitive strengths could occur as part of existing information gathering practices: “*Maybe... it’s more something that we just need to hold in mind and have it as part of our getting to know the young person as we move through the initial stages of working with them*” (P7). However, some clinicians were concerned that assessing cognitive strengths without a formal structure could lead to inaccurate appraisals of strengths or that a deficit focus would be inadvertently adopted: “*I mean at the moment, it’s sort of ad hoc [appraisal of cognitive strengths], by what’s sort of presenting in the room. Certainly, it’s more ... more the deficits that come to people’s attention*” (P1).

Several clinicians advocated assessing cognitive strengths via a task-based process that focused on how people functioned while completing certain operations. Clinicians who supported this approach argued that it had more relevance to aiding a young person’s functional recovery:

“*I think you get so much more out of doing a functional sort of based assessment with someone as opposed to sort of just, ‘Here’s a question, can I have an answer?’ I think it’s really ... a dynamic way of finding about um, how clients sort of operate in the day-to-day.*” (P2).

Others suggested a combined approach where information on cognitive strengths would be gathered from informants (e.g., family and schools), in addition to some form of self-assessment.

## Discussion

The present study aimed to capture clinicians’ insights and perspectives regarding the conceptualisation, percieved benefits and risks, and considerations for development of assessment and treatment methods focused on cognitive strengths in FEP. This qualitative account contributes to a broader investigation of the perceived acceptability and utility of a cognitive strengths approach amongst various stakeholders (experts, clinicians, and young people) within the FEP service context. Five overarching themes summarised clinicians’ accounts: (1) pro-strengths attitude despite unfamiliarity and minimal use, (2) default to a cognitive deficit lens, (3) potential benefits of a cognitive strengths approach, (4) potential risks and barriers, and (5) considerations for successful implementation.

### Summary and implications

Based on the current sample, clinicians were receptive to exploring a cognitive strengths-based assessment and treatment for future development. This response is promising as positive attitudes are a necessary first step for successfully implementing novel treatment approaches [26]. However, some clinicians struggled to conceptualise cognitive strengths and inadvertently defaulted to a deficit lens, suggesting that a cognitive strengths focus was limited or non-existent in current practice. Consequently, current treatment approaches to FEP may not tailor best to young people’s recovery goals [6]. The limited understanding of cognitive strengths in practice amongst clinicians is consistent with the views of expert researchers [23], demonstrating a need to explore a novel approach within the field collectively.

Clinicians identified several potential benefits of a cognitive strengths approach in FEP that aligned with those found in broader strengths approaches These benefits (e.g., self-efficacy) mediate the relationship between cognition and real-world functioning (e.g., social functioning) [16, 27] and directly enhance cognitive performance [28]. Clinicians also believed that a cognitive strengths approach would reduce self-stigmatisation and perceived defectiveness in young people. Both constructs are known to negatively impact functional and personal recovery [29, 30]. Furthermore, developing knowledge of a young person’s cognitive strengths was believed to assist clinicians and young people to navigate functional recovery pathways together. This collaboration toward shared goals is considered essential for strengthening the therapeutic alliance [31] and reflects the link between self-competence and intrinsic motivation underlying the self-determination theory [32]. Thus, awareness of cognitive strengths could be instrumental in transforming current biomedical-centric approaches towards more functional and personal recovery focused services.

Clinicians were concerned that a cognitive strengths approach could neglect addressing young people’s deficits. Indeed, understanding and treating deficits in FEP is essential to treatment and underpins diagnosis, illness phase, and preventing relapse, and is recommended in national best-practice guidelines [7, 33]. Nevertheless, strengths-based approaches do not seek to dismiss the treatment of deficits but do aim to identify and utilise strengths for therapeutic gain [17, 34]. Some clinicians expressed concern that young people might feel unacknowledged if cognitive strengths were emphasised. This concern is worthy of consideration, as feeling misunderstood by mental health professionals has been identified as a significant source of distress amongst FEP service users [35]. Notwithstanding the need for sensitive management of this concern, reviews of strengths-based approaches have found that FEP consumers do feel acknowledged [36].

Taking a strengths-based view to cognition was novel for clinicians, although this is not surprising given that the approach has not been previously researched or disseminated [6, 23]. Nevertheless, clinicians’ practical recommendations offer an idea of how to address the concerns during future development and implementation. One key recommendation was to communicate to young people a clear benefit and tangible goal for the assessment or treatment by using scenarios or examples that closely resemble real-world functioning. These suggestions address previously raised concerns that many cognitive assessments bear limited ecological validity in how cognitive performance is used in everyday situations [28]. Furthermore, to alleviate time concerns and the training burden that is common in recovery-focused mental health care [37], several clinicians suggested that a specialist clinician should implement the cognitive strengths assessment or treatment. However, such a proposal could complicate implementation. Obtaining the perspectives of young people with FEP will be important for determining the most acceptable approach to assessing cognitive strengths.

### Limitations

The responses may be susceptible to sampling bias, given that participants were drawn from a single FEP service. Attempts were made to gather a range of perspectives from different clinicians from various backgrounds and years of experience, yet the results may not reflect the experiences of other FEP service contexts. While clinicians who work at EPPIC do not formally practice strengths-based approaches (e.g., positive psychology), many are likely to be aware of the strength and recovery focus that is promoted at an organisational level. Such knowledge may have influenced the opinions of clinicians about the study’s central proposal. This study also considered a cognitive strengths approach at a pre-development stage. Thus, clinicians were unable to provide insight toward a tangible assessment or treatment procedure. Finally, three clinicians in this study (25% of the sample) were previously involved in research exploring cognitive function in young people with FEP. Awareness of cognition in FEP and its impact of functioning, alongside potential challenges associated with other evidence-based treatments (e.g., cognitive remediation), may have contributed to the enthusiasm regarding a cognitive strengths approach.

## Conclusions

The perspectives gathered from clinicians are critical for the future design and implementation of a cognitive strengths-based assessment and treatment. However, future studies will need to establish whether a cognitive strengths approach to addressing cognition in FEP does improve the therapeutic alliance, self-efficacy, and translates to real-world functioning. While this study has provided promising perspectives from clinicians regarding cognitive strengths-based approaches in FEP, consultation with young people with lived experience is essential and must be explored in future research.

## Supplementary Information

**Additional file 1.**

**Additional file 2.**

## Data Availability

The qualitative data used in and analysed during the current study cannot be made publicly available for confidentiality reasons, but they can be discussed with the corresponding author on reasonable request.

## Abbreviations

CR: Cognitive Remediation
EPPIC: Early Psychosis Prevention and Intervention Centre
FEP: First Episode Psychosis
OYH: Orygen Youth Health
