# Supplementary material for: Cognitive strengths in first episode psychosis: a thematic analysis of clinicians’ perspectives
Source: BMC Psychiatry. 2021 Dec 8;21:612. doi: 10.1186/s12888-021-03627-y (PMC8653580; doi:10.1186/s12888-021-03627-y)
Supplement: Supplementary file 1 — Additional file 1. [file 12888_2021_3627_MOESM1_ESM.docx]

**Supplementary Material**

**Semi-Structured Interview Questions**

**Clinician Interview Questions**

Demographic Questions: Age, Gender, Qualifications, Area of Expertise and years of working in the field.

Cognitive Strengths Questions:

1. What do you think of when you hear the term “cognition”?
2. Do you consider cognitive strengths in your field of work? If so, tell me about how you conceptualise them? (Prompt: if unfamiliar with the concept of cognitive strengths: Cognitive strengths can be conceived of in a variety of ways. For example, they can be thought of as more traditional thinking skills; as abilities that are required for social interactions; or, as practical skills relevant to a particular task or circumstance.) Another way we’ve thought about it….
3. In thinking about cognitive strengths, what are some other examples that come to mind?
4. The existing clinical and treatment literature on first-episode psychosis focuses on impairments to cognitive function. Do you think there has been enough focus on cognitive strengths?
5. What comes to mind when you think of cognitive strengths in early psychosis specifically?
6. Which cognitive strengths would relate to social functioning?
7. Which cognitive strengths would relate to general functioning, including vocational functioning?
8. How do you think cognitive strengths could be used to enhance social and general functioning, including vocational functioning *(Prompt: e.g., awareness of strengths - knowledge of practical application of strengths - increased self-efficacy - functional improvement)?*
9. How do you think cognitive strengths could be assessed? (Prompt: eg, self-report, clinician rated, objective assessment)

*(Preface: One of the main aims of this study is to gain diverse perspectives about what cognitive strengths might be to inform the later development of an assessment and intervention.)*

1. How could such an assessment of cognitive strengths be user friendly?
2. How do you think such an assessment could be clinically useful?
3. How do you think young people would respond to a cognitive strengths-based assessment?
4. How do you think assessing cognitive strengths could be incorporated into treatment for first-episode psychosis?
5. Do you think there would be benefits to including a cognitive strengths-based intervention into the treatment of first-episode psychosis? If so, what might these benefits be?
6. Can you think of any foreseeable barriers to using a cognitive strengths-based intervention with the first-episode psychosis population? What about adverse effects?
7. Can you think of any downsides to focusing on cognitive strengths?
