# Supplementary material for: Cognitive strengths in first episode psychosis: a thematic analysis of clinicians’ perspectives
Source: BMC Psychiatry. 2021 Dec 8;21:612. doi: 10.1186/s12888-021-03627-y (PMC8653580; doi:10.1186/s12888-021-03627-y)
Supplement: Supplementary file 2 — Additional file 2. [file 12888_2021_3627_MOESM2_ESM.docx]

**Table.** Cognitive strengths identified by clinicians

| Cognitive Strengths | Number of References |
| --- | --- |
| Memory | 7 |
| Conversational ability | 6 |
| Working memory | 6 |
| Reasoning ability | 5 |
| Ability to engage with people | 4 |
| Problem solving | 4 |
| Ability to retain and organise information | 3 |
| Concentration | 3 |
| Organisational skills | 3 |
| Processing speed | 3 |
| Ability to be flexible in thinking | 2 |
| Ability to creatively cope with challenges | 2 |
| Cognitive flexibility | 2 |
| Decision making | 2 |
| Executive function | 2 |
| Intelligence | 2 |
| Planning | 2 |
| Problem solving for residual symptoms | 2 |
| Receptive and expressive language skills | 2 |
| Resiliency factors | 2 |
| Self-reflection | 2 |
| Social Cognition | 2 |
| Theory of Mind | 2 |
| Ability to acquire knowledge | 1 |
| Ability to apply creative thinking | 1 |
| Ability to apply memory practically | 1 |
| Ability to complete a task you enjoy | 1 |
| Ability to consider alternatives | 1 |
| Ability to consider different behavioural options | 1 |
| Ability to complete a task you enjoy | 1 |
| Ability to independently complete a task | 1 |
| Ability to think through a task | 1 |
| Ability to understand others’ perspectives | 1 |
| Ability to weigh up risks | 1 |
| Adaptable | 1 |
| Attention | 1 |
| Behavioural control | 1 |
| Being a good historian | 1 |
| Cognitive flexibility outside of delusions | 1 |
| Coherent recollection of past events | 1 |
| Collaborative thinking ability | 1 |
| Complex comprehension ability | 1 |
| Confident completion of a task | 1 |
| Coping skills | 1 |
| Focused attention | 1 |
| Good at remembering names | 1 |
| Good storytelling ability | 1 |
| Higher-order processing | 1 |
| Initiating tasks or activities | 1 |
| Insight into problems | 1 |
| Language skills | 1 |
| Non-binary thinking style | 1 |
| Perceptual reasoning | 1 |
| Perspective taking | 1 |
| Pre-morbid cognitive functioning | 1 |
| Responsiveness | 1 |
| Skill acquisition | 1 |
| Skilled in 'hands-on' activities | 1 |
| Thought identification | 1 |
| Verbal working memory | 1 |
| Work through difficult concepts | 1 |
